# VALIDATE: Exploiting the synergy between complex intracellular pathogens to expedite vaccine research and development for tuberculosis, leishmaniasis, melioidosis and leprosy

**DOI:** 10.12688/f1000research.14386.1

**Published:** 2018-04-23

**Authors:** Helen A. Fletcher, Mitali Chatterjee, Andrea Cooper, Tracy Hussell, Paul M. Kaye, Joann Prior, Rajko Reljic, Samantha Vermaak, Martin Vordermeier, Ann Williams, Helen McShane

**Affiliations:** 1 The London School of Hygiene and Tropical Medicine, London, UK; 2 Department of Pharmacology, Institute of Postgraduate Medical Education & Research, Kolkata, India; 3 Department of Infection, Immunity and Inflammation, University of Leicester, Leicester, UK; 4 Manchester Collaborative Centre for Inflammation Research, University of Manchester, Manchester, UK; 5 Centre for Immunology and Infection, University of York, York, UK; 6 Defence Science and Technology Laboratory, Porton Down, UK; 7 Institute for Infection and Immunity, St George's University of London, London, UK; 8 The Jenner Institute, Nuffield Department of Clinical Medicine, University of Oxford, Oxford, UK; 9 Department of Bacteriology, Animal and Plant Health Agency, Weybridge, UK; 10 National Infection Service, Public Health England, Porton Down, UK

**Keywords:** Tuberculosis, TB, vaccine, leishmaniasis, leprosy, melioidosis, neglected, intracellular

## Abstract

For several complex intracellular pathogens, we have an urgent need for effective vaccines and yet there are common barriers to vaccine development. These diseases, including tuberculosis, leishmaniasis, leprosy and melioidosis, cause a huge burden of disease and disproportionately affect low and middle income countries. They are therefore often neglected due to the marginalisation of affected populations and the poor predicted commercial return on investment. Barriers to vaccine development include an incomplete understanding of protective immunity and translation from the bench into clinical vaccine trials. The current linear approach to vaccine research and development for these pathogens, which involves basic research, vaccine design, and vaccine evaluation in preclinical challenge models and clinical trials, is inefficient for these complex intracellular pathogens. We have established a Global Challenges Research Fund Network for VAccine deveLopment for complex Intracellular neglecteD pAThogEns, “VALIDATE”, where we aim to adopt a more flexible, integrated cross-pathogen approach to accelerate vaccine research and clinical development for these four pathogens, by cross-pathogen analyses, cross-discipline collaborations, and repeated integration of data from human and animal studies.

This network provides a unique opportunity to bring together individuals working on four exemplar complex intracellular neglected pathogens ( *M.tb*, *Leishmania* spp., *B. pseudomallei* and *M.leprae*), which share a common lifestyle as pathogens of macrophages, induce similar end-stage pathologies and alter host immune and metabolic responses. The horizontal collaborations established throughout this network, together with the provision of a protected environment for early data sharing, will exploit these biological synergies.  By interrogating mechanisms that lead from infection to disease, we will be able to develop common vaccine development strategies for these and other complex intracellular pathogens.

## Introduction

The global burden of disease and death caused by *Mycobacterium tuberculosis* ( *M.tb*), *Leishmania* spp, *Burkholderia pseudomallei* and *M.leprae* is enormous. Tuberculosis (TB) kills more people than any other infectious disease, with 1.7m deaths and 10.4m new cases in 2016 ^1^. The significant economic impact of TB in low- and middle income countries (LMICs) is due to the disproportionate involvement of the most economically active young adults. Furthermore, bovine TB has a very significant effect on health and economic development in LMIC ^2, 3^. The leishmaniases represent a group of heterogeneous diseases caused by intracellular protozoan parasites of the genus *Leishmania*. They are recognized by the WHO as major neglected diseases of poverty, and disproportionately affect populations in LMICs. Approximately 1.5m new cases occur each year, across 98 countries worldwide, with 20,000–40,000 deaths ^4^. Canine visceral leishmaniasis is not only a veterinary problem but a significant reservoir for human disease, notably in Brazil and in countries bordering the Mediterranean. As with TB, leishmaniasis and poverty exist in a vicious circle, with the economic impact for patients, families and communities well documented.

Leprosy and melioidosis also compromise economic productivity in LMICs, are difficult to treat and are in need of effective vaccination strategies. In 2016, there were 216,108 new leprosy cases registered globally ^5^. 14 countries contain 95% of these globally reported cases, all of which are in LMICs. Of these, India has the greatest number of cases (59%), followed by Brazil (14%) and Indonesia (8%). Although the number of cases worldwide continues to fall, pockets of high prevalence remain in certain areas such as Brazil, South Asia (India, Nepal, Bhutan), some parts of Africa (Tanzania, Madagascar, Mozambique), and the western Pacific. Melioidosis is a disease caused by the Gram-negative soil-dwelling bacterium *Burkholderia pseudomallei* ^6, 7^. The estimated global burden of melioidosis is 165,000 human melioidosis cases/pa, causing 89,000 deaths ^7^. The burden of disease is in South East Asia, where the in-hospital mortality is ~40% ^8^. These estimates of incidence for leprosy and melioidosis are likely to be significant under-estimates. The development of effective vaccines against any or all of these pathogens would have a significant health benefit around the world.

## What does VALIDATE aim to achieve?

The lack of rapid, coordinated dissemination of information between research groups hinders vaccine development. Our strategy is to establish a network of multi-disciplinary scientists across the UK and LMICs, who work on vaccine research and development for these four complex intracellular neglected pathogens. We will take an interdisciplinary approach with immunologists, clinicians, social scientists, veterinarians, epidemiologists, bioinformaticians, mathematical modellers, and animal model experts to overcome barriers to progression of vaccine development. The coordinated, integrated and iterative sharing of data to define protective immunity in animal models, in target animal species, and in human experimental medicine studies, across complex intracellular pathogens will expedite the development of effective vaccines. Our particular focus is on building and strengthening cross-pathogen, cross-species, cross-discipline and cross-continent collaborations, to foster novel insights and new perspectives that lead to an increased understanding of the nature of protective immunity. Currently, few horizontal collaborations occur between these distinct pathogen research fields. VALIDATE will add value by establishing horizontal collaborations whereby innovative research solutions in one field will be rapidly disseminated to the other fields. This interaction will leverage progress in one field and promote accelerated identification of immune mechanisms of protection and evaluation of vaccine candidates in human and animal models. Given the many common immunological and microbiological features of the complex intracellular pathogens selected as the focus of VALIDATE, these newly-formed horizontal cross-pathogen collaborations will yield novel insights that will be explored iteratively in *in vitro* and *in vivo* experiments in animal models and human experimental medicine studies. For example, a key feature of both TB and leishmaniasis is granuloma formation. Imaging and transcriptomics have been used to define the *Leishmania* granuloma; these techniques could be further used to interpret the TB granuloma ^9, 10^.

To date, VALIDATE has 114 members from 24 countries, 17 of these LMICs, and this range of knowledge and experience will broaden our scope and ensure relevance. The VALIDATE members are selected for their complementary scientific expertise and range from internationally recognised leaders in their field to Early Career Researchers (ECRs), with students and members of the general public who are interested in vaccine research also welcome. All scientific members have significant track records in research into TB, leishmaniasis, leprosy or melioidosis. Members can come from academia, governmental organisations, non-profits, and industry. Specific fields of interest include basic immune mechanisms in humans and preclincial animal models, mucosal immunity, immune correlates of protection, immunopathogenesis, translational vaccine development, mathematical modelling and social science aspects of vaccinology.

## How will we do it?

VALIDATE has four main activity streams.

Firstly, we are providing relevant funding to our members: as pump-priming grants of £20–50k for innovative collaborative research projects; training grants to enable our ECRs to attend courses, workshops and laboratory exchanges useful for their career progression; and as ECR Fellowships to springboard these Fellows to scientific independence as new group leaders.

Secondly, VALIDATE is developing a dedicated data-sharing portal for members only, which will encourage real-time sharing of data, catalysing the application of insights from one field into another. The VALIDATE Research Data Analyst is tasked with actively searching for data synergies and differences where researchers working on different pathogens can learn from another, and works with members on existing and arising datasets.

Thirdly we provide Continuing Professional Development (CPD) opportunities for our members, including workshops on areas of mutual interest, seminars (that are live-streamed online so that overseas members can also benefit), and a mentoring scheme for ECRs and early PIs open to members across the world – we have linked five mentees with their chosen mentors in our first round. Scientific research is a valuable economic activity to the host country, and we will build sustainable human resource capacity within both the UK and low- and middle-income member countries.

Finally, we are developing a vibrant and interactive network, facilitating the formation of new collaborations and ideas, and speeding the dissemination of useful information amongst our members using the full range of communications tools. We have created a hub website ( www.validate-network.org) where our members can easily find information about new research and papers, relevant funding calls, events, and training, mentoring and other opportunities of interest. Interested parties can read about our funded work to date, while a searchable directory of members on our website is facilitating the formation of new collaborations. Our social media ( @NetworkValidate) raises awareness of these four pathogens and VALIDATE’s research through engagement of members, other scientists and the lay public. An annual meeting, free to all members, furthers our outreach, and boosts existing and potential collaborations. There are travel scholarships available for up to seven LMIC members each year to facilitate the broadest level of attendance.

## Summary

VALIDATE provides a unique opportunity for an interdisciplinary and integrated approach to vaccine research and development for these four exemplar complex intracellular neglected pathogens. Sustainable collaborations are being formed and strengthened by the resources provided, innovative research has been funded, and ECR progression is underway. We are using this platform to accelerate vaccine development for our focus pathogens, and are building our funding portfolio to ensure sustained progress in these critical areas.

## Data availability

No data is associated with this article.
