# Supplementary material for: VALIDATE: Exploiting the synergy between complex intracellular pathogens to expedite vaccine research and development for tuberculosis, leishmaniasis, melioidosis and leprosy
Source: F1000Res. 2018 Apr 23;7:485. [Version 1] doi: 10.12688/f1000research.14386.1 (PMC5974572; doi:10.12688/f1000research.14386.1)
Supplement: Peer review history

# Peer review: Referee response for version 1

**DOI:** 10.5256/f1000research.15655.r34153

**Published:** 2018-06-26

**Authors:** Steven G. Reed

**Affiliations:** 1 Infectious Disease Research Institute, Seattle, WA, USA

Mechanisms of immunity to intracellular pathogens, such as those causing tuberculosis, leishmaniasis, and leprosy have much in common, and as such knowledge in each of these areas should be leveraged and applied as much as possible to these and other intracellular pathogens. The functional definition of CD4 T cell subsets was pioneered in experimental leishmaniasis models and the knowledge has been applied to other infectious diseases, allergy and cancer. Of particular relevance is the selection of vaccine delivery systems and adjuvants that may selectively drive desired immune responses.

I have read this submission. I believe that I have an appropriate level of expertise to confirm that it is of an acceptable scientific standard.

# Peer review: Referee response for version 1

**DOI:** 10.5256/f1000research.15655.r34293

**Published:** 2018-05-29

**Authors:** Lisa A. Morici

**Affiliations:** 1 Tulane University School of Medicine, Department of Microbiology and Immunology, New Orleans, LA, USA

This article comprehensively describes the scope and purpose of VALIDATE, a Global Challenges Research Fund Network. VALIDATE aims to exploit the similarities among complex intracellular bacterial pathogens, including those responsible for tuberculosis, leishmaniasis, melioidosis, and leprosy. Of the target diseases, only tuberculosis receives considerable attention from major funding agencies. Establishment of VALIDATE will help unite researchers from across the globe to accelerate vaccine development against these largely neglected diseases. It will also provide a resource for data sharing, pilot funding, and mentorship for young investigators.

I have read this submission. I believe that I have an appropriate level of expertise to confirm that it is of an acceptable scientific standard.

# Peer review: Referee response for version 1

**DOI:** 10.5256/f1000research.15655.r33590

**Published:** 2018-05-02

**Authors:** Antonio Campos-Neto

**Affiliations:** 1 Detectogen Inc., Westborough, MA, USA

The article reports the goals of the recently created initiative named VALIDATE, which is an organization funded by the Global Challenges Research Fund (GCRF) Networks in Vaccines Research and Development, which is co-funded by the The Medical Research Council (MRC) and The Biotechnology and Biological Sciences Research Council (BBSRC).

 VALIDATE is a fantastic Network that aims to promote vaccine research and development for complex intracellular pathogens that cause significant disease burden in low and middle-income countries (LMIC).  The authors’ summary of VALIDATE activities is outstanding.

 The authors and founding members of this much needed initiative deserve the compliments of scientific communities and Government authorities of not only of LMICs but also of developed countries.

 One minor suggestion is to add in the article a sentence or two indicating the plans for long term funding sustainability of VALIDATE.

I have read this submission. I believe that I have an appropriate level of expertise to confirm that it is of an acceptable scientific standard.
